# Hemodialysis and hemodiafiltration differently modulate left ventricular diastolic function

**DOI:** 10.1186/1471-2369-14-76

**Published:** 2013-04-02

**Authors:** Árpád Czifra, Alida Páll, Julianna Kulcsár, Kitti Barta, Attila Kertész, György Paragh, István Lőrincz, Zoltán Jenei, Anupam Agarwal, Abolfazl Zarjou, József Balla, Zoltán Szabó

**Affiliations:** 1 Department of Medicine, Division of Nephrology, Medical and Health Science Center, University of Debrecen, Pf. 19., Nagyerdei krt. 98, Debrecen 4012, Hungary; 2 Department of Cardiology, Medical and Health Science Center, University of Debrecen, Debrecen, Hungary; 3 Department of Medicine, Nephrology Research and Training Center and Center for Free Radical Biology, University of Alabama at Birmingham, Birmingham, AL, USA

**Keywords:** Hemodiafiltration, Hemodialysis, Echocardiography, Diastolic function, Nitric oxide

## Abstract

**Background:**

Renal replacement therapy may have a favorable effect on diastolic left ventricular function, but it is not clear whether hemodiafiltration is superior to hemodialysis in this field. Nitric oxide (NO) and asymmetric dimethylarginine (ADMA) may play a role in the changes of intracardiac hemodynamics, but it is not clear whether the different renal replacement methods have disparate influence on the metabolism of these materials.

**Methods:**

Thirty patients on renal replacement therapy were investigated. First, data was analyzed while patients received hemodiafiltration over a period of three months. Then, the same patients were evaluated during treatment with hemodialysis for at least another three months. Echocardiography was performed before and after renal replacement therapy.

**Results:**

No significant difference was found in the volume removals between hemodialysis and hemodiafiltration. The left atrial diameter and transmitral flow velocities (E/A) decreased significantly only during hemodiafiltration. A positive correlation was observed between the left atrial diameter and E/Ea representing the left ventricular pressure load during hemodiafiltration. Significant correlations between NO and A and E/A were observed only in the case of hemodiafiltration.

**Conclusion:**

Hemodiafiltration has a beneficial effect on echocardiographic markers representing left ventricular diastolic function. This could be attributed to the differences between the dynamics of volume removal and its distribution among liquid compartments.

## Background

Cardiovascular disorders remain the leading cause of mortality in chronic kidney disease (CKD) patients [1]. Despite significant advances in renal replacement therapy techniques, the cardiovascular mortality rate of these dialyzed, accounts for more than 50% of the total mortality rate and its frequency is 17 times higher compared to the healthy population. Heart failure plays an important role in the unfavorable changes in mortality statistics, which accounts for 64% at the time of initiation of dialysis [2]. According to a recent study the median survival rate of 62 months among hemodialyzed patients decreased to 36 months when heart failure was present [3]. The most common causes of heart failure in patients with chronic kidney disease are advanced age, female sex, hypertension, diabetes mellitus and atherosclerosis, ischemic and structural heart disease. Renoparenchymal hypertension is also an important factor, the frequency of which, in patients receiving renal replacement therapy, is estimated to be 89% [4]. The adaptive mechanisms induced by the nephrons’ hyperfiltration, and the harmful effects of persistent high blood pressure caused by hypervolemia, have a major effect in the development of left ventricular hypertrophy, which can also lead to ventricular filling disorder. Furthermore, endothelial dysfunction plays an important role in the genesis of atherosclerosis and hypertension in this group of patients. Nitric oxide (NO) and the competitive inhibitor of nitric oxide synthase - asymmetric dimethylarginin (ADMA) - are known to be the key mediators in the regulation of vascular tone. Altered bioactivity of nitric oxide and an enhanced formation of oxygen-derived free radicals were shown to be present in patients on chronic hemodialysis [5]. It is important to note that based on the severity of diastolic heart function, patients may be relatively asymptomatic. Nevertheless, regardless of clinical manifestation, diastolic heart failure is an independent risk factor for cardiovascular mortality in both symptomatic and asymptomatic patients. Furthermore, diastolic dysfunction is an important underlying factor in the development of atrial fibrillation [6] and the pathomechanism is postulated to be due to increased atrial volume load. Evidence suggests that the mortality rate in patients participating in hemodiafiltration programs is 35% lower than in those receiving conventional hemodialysis [7]. Whereas conventional treatment eliminates uremic toxins, depending on their molecular weights, by diffusion, hemodiafiltration also eliminates the medium molecular weight toxic polypeptides (characterized by β-2 microglobulin) by convective transport [8]. Whether hemodiafiltration and conventional hemodialysis affect the left ventricular diastolic function and vascular tone mediators differently is not yet clear. Therefore, the extent of favorable mortality rates in hemodiafiltration cases that may be attributed to the diastolic function of the myocardium and the changes in intracardiac pressure relations remains to be elucidated.

## Methods

The statistical analysis was carried out with the help of the SAS 8.2 for Windows software. The variations of the investigated parameters over time and the difference between the two modalities were investigated by using repeated measures ANOVA. The correlation between the parameters was analyzed by using the Pearson’s test when the distribution was normal and by Spearman’s rank test in the case of not normal distribution. Throughout the analysis the p < 0.05 probability level was considered statistically significant. Data is presented as mean ± SD. The examination results of thirty non-diabetic patients, with end stage renal failure were studied (18 males, 12 females, mean age 60 ± 13.6 years). At the time of selecting patients for the study the mean time of renal replacement therapy was 93.13 ± 70.09 months. Firstly, we collected and analyzed the data from the patients while they received hemodiafiltration, and then from the same patients during treatment with conventional hemodialysis for at least three months. This was followed, at the time of the regular hemodialysis, by further data collection and investigation. Exclusion criteria were: any impulse generation or conduction disease which could affect echocardiographic findings, or makes it impossible to measure certain echocardiographic parameters. Thus, atrial fibrillation, where no atrial contraction appears and A wave cannot be detected during Doppler examination was an exclusion criterion. In the case of atrial flutter the evaluation of late diastolic transmitral velocity may not be easy to measure, so patients with this type of arrhythmia were also excluded. Furthermore, patients with amyloidosis, sarcoidosis, carcinoid, hemochromatosis, and pericardial constriction were not involved in the study, also patients with pseudonormalization pattern were excluded. Patients were included who were suffering from end stage kidney disease (Stage 5) participating in regular hemodialysis program in our center, and were willing to give their informed consent to take part in the study. The chronic kidney disease of the studied population was caused by the following: chronic glomerulonephritis (n = 5), hypertensive and vascular nephropathy (n = 12), chronic pyelonephritis (n = 1), polycystic kidney disease (n = 2), analgesic nephropathy (n = 3), renal agenesis (n = 1), systemic lupus erythematosus (n = 2), and vasculitis (n = 4). Ninety percent of the patients suffered from hypertension (arterial blood pressure requiring antihypertensive therapy > 140/90 mmHg), 16.7% had hypercholesterolemia (serum cholesterol >5.2 mmol/L) and 10% had ischemic heart disease as shown by stress test. The patients – after receiving detailed information about the trial – confirmed, in writing, their will to participate in the study, and the Ethics Committee of the University of Debrecen approved the study protocol. All procedures and treatments used on the study group were part of standard care. Before and after the renal replacement therapy transthoracic echocardiography investigations (M-mode, 2 D) were performed with a pulsed, and a continuous wave and tissue Doppler technique (Philips ATL HDI 5000 imaging system with a 3.5 MHz transducer). During the examinations the left atrium’s cross diameter was measured from the parasternal long-axis view, then, based on an apical four-chamber view, the Simpson’s method was used to determine the left ventricular ejection fraction. Using the Devereux-Reichek formula the left ventricular mass index was calculated ({1.04x[(end-diastolic diameter of the left ventricle + interventricular septum thickness + the posterior wall thickness of the left ventricle)^3^-end-diastolic diameter of the left ventricle^3^] -14}/height). The early diastolic transmitral peak flow velocity (E) was determined from the apical four-chamber view with the help of a pulsed-wave Doppler, and the peak flow velocity in the late diastolic period during the atrial contraction (A) was also determined. The time between the beginning and the end of the E-wave deceleration slope was defined as deceleration time (DT). Using Tissue Doppler Imaging (TDI) we evaluated the early diastolic velocity of the septal mitral annulus (Ea) from the apical approach. In order to estimate the left ventricular filling pressure we calculated the ratio of the E and Ea (E/Ea). First degree (mild) diastolic dysfunction (DD) was defined as: E/A < 0.8, E/Ea < 8, DT > 200 msec and second degree (medium) DD as: 0.8 < E/A < 1.5, 8 < E/Ea < 13 and the DT is 160–200 msec. The criteria for third degree (severe) DD were: E/A > 2, E/Ea > 13, DT < 160 msec. At the time of randomization 7 of our patients belonged to the severe group, 15 belonged to the medium, and 8 to the mild group. In order to differentiate between the normal and pseudonormal pattern, where E/A ratio was proved to be normal (between 1–1.5) and deceleration time was found to be above 140 msec, the possible pseudonormalization was evaluated with the measurement of E/Ea ratio, furthermore Valsalva maneuver was performed. Patients with pseudonormalization (relaxation pattern occurring during Valsalva maneuver and E/Ea > 10) were not involved in the study. The diameter of the inferior vena cava was measured from the subcostal view. Hemodialysis and hemodiafiltration were performed three times a week during 4-hour long sessions with Fresenius 4008 S and H devices (Fresenius Medical Care, Bad Homburg, Germany), with Fx60 and Fx80 high-flux polysulfone dialysis capillaries (Fresenius). During hemodiafiltration 15.16 ± 5 liters of ultrafiltrate was removed. The substitution fluid was prepared on-line from dialysis solution through a set of two membranes to purify it before infusing it directly into the blood line. The replacement solution was manufactured on-line from ultrapure water and consisted of 138 mmol/L sodium, 2 or 3 mmol/L potassium (in 13 cases 2 mmol/l in 17 cases 3 mmol/l), 1.5 mmol/L calcium, 0.5 mmol/L magnesium, and 1 g/L glucose. The blood flow was 338 ± 11.6 ml/min and did not differ significantly during the respective procedures (p < 0.05). The bicarbonate dialysis solution contained 138 mmol/L sodium, 2 or 3 mmol/L potassium (the same as the substitution solution), 1.5 mmol/L calcium, 0.5 mmol/L magnesium, and 1 g/L glucose. During the treatment no drugs other than isotonic sodium chloride and sodium heparin solutions were administered. The previous drug therapy (digitalis, nitrates, beta-blockers and calcium channel blockers, angiotensin converting enzyme inhibitors, and angiotensin receptor blockers) remained unchanged. The arterial blood pressure was monitored non-invasively. In order to monitor the heart rhythm and to analyze the RR cycle length characterizing the heart frequency, five conventional 12-lead electrocardiograms were carried out in each case: at the beginning of the treatment, in the 15th, 30th^,^ and the 240th minutes, and finally two hours after the treatment. The ECG results were recorded by a 12-channel ECG device at 25 mm/sec recording speed (Hewlett Packard Page Writer 200i), and during the recordings the patients were in the supine position, breathing freely, and were not talking. Nitric oxide concentration of the serum was determined by the modified method of Navarro-Gonzalez and ADMA concentrations in the plasma were measured using an enzyme-linked immunosorbent assay (ELISA).

## Results

**Figure 1:**
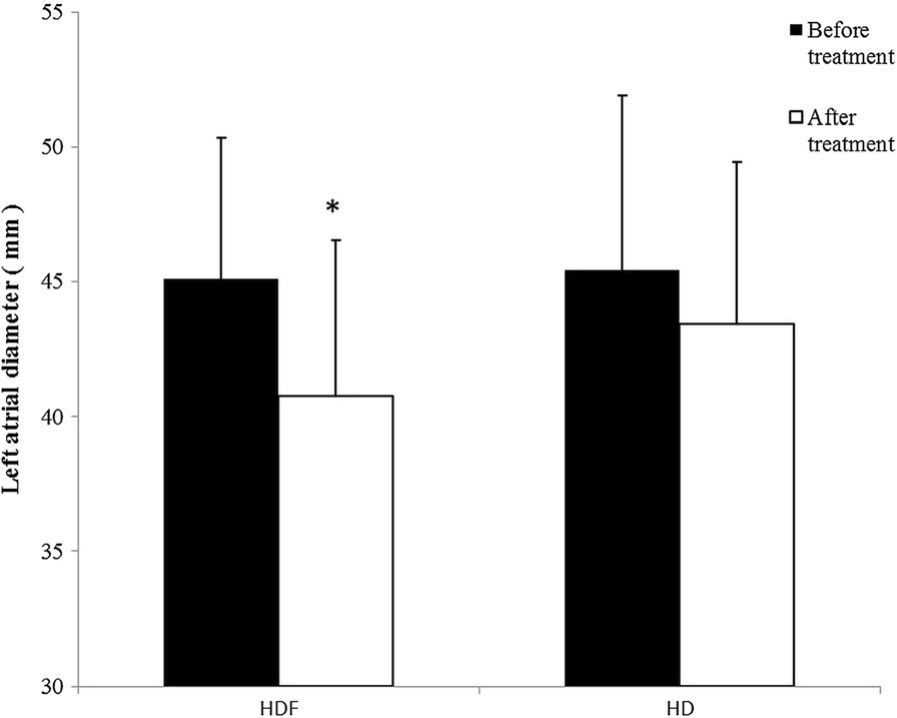
**Changes in left atrial cross diameter during the sessions.** A significant decrease in left atrial cross diameter occurred during hemodiafiltration (p < 0.05), but the change did not prove to be significant in the case of hemodialysis. HD: hemodialysis, HDF: hemodiafiltration (*p < 0.001).

**Figure 2:**
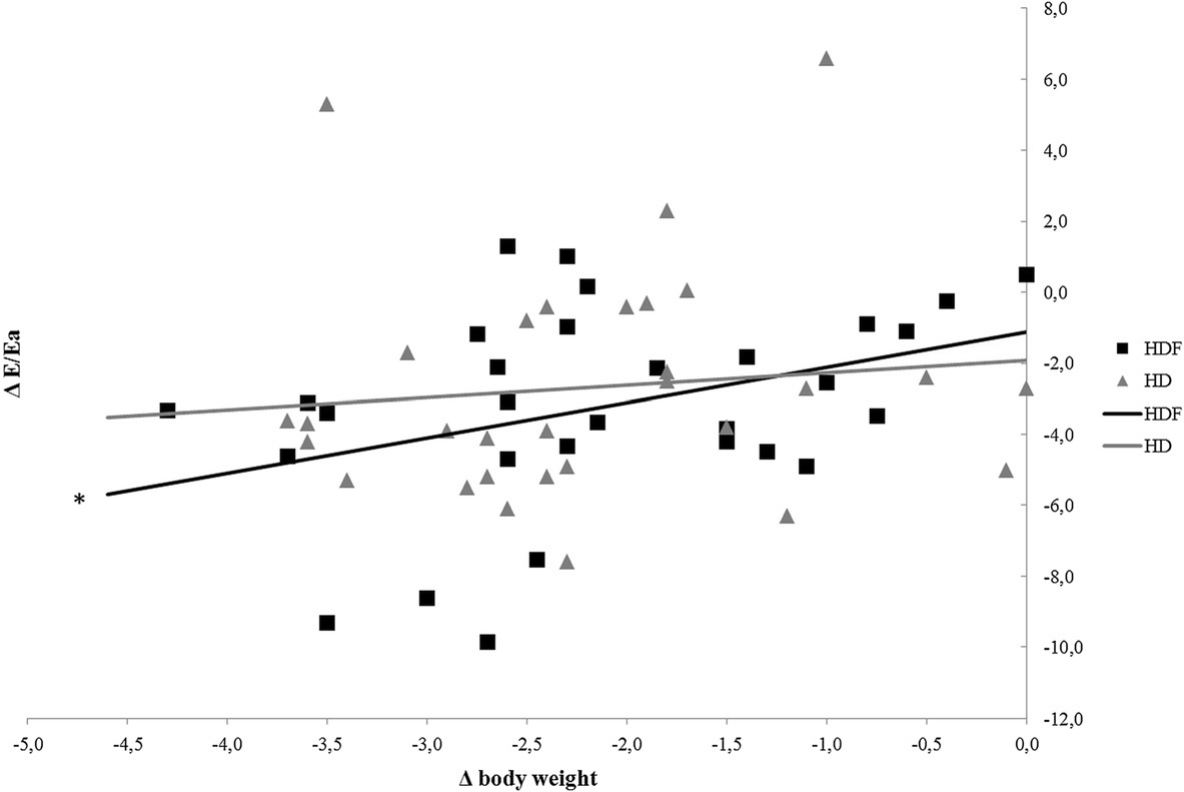
**The correlation between the changes in body weight and E/Ea ratio in the case of the two renal replacement modalities.** A significant correlation was observed only in the case of hemodiafiltration. HD: hemodialysis, HDF: hemodiafiltration (*p = 0.041).

**Figure 3:**
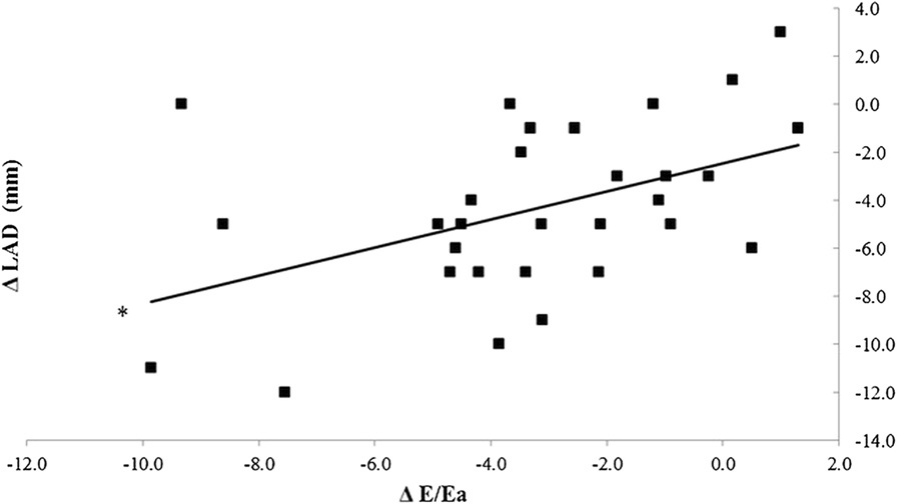
**The correlation between the left atrial cross diameter and the decrease in E/Ea ratio during hemodiafiltration.** The decreasing atrial diameter was significantly correlated with the decrease in E/Ea. ΔLAD: changes in the left atrial cross diameter (*p = 0.013).

A significant decrease in left atrial cross diameter (p < 0.001) occurred during hemodiafiltration which was not observed in the case of hemodialysis (p = 0.11) (Figure 1). Echocardiography revealed that E and E/Ea decreased in both renal replacement modalities (p < 0.001), however, no statistically significant difference was found between the two renal replacement methods (p = 0.37). Regarding E/Ea measured before the beginning of the sessions we did not find statistically significant differences either (12.56 ± 3.56 vs. 11.65 ± 4.49, p = 0.14). E/A ratio’s significant decrease was observed only during hemodiafiltration (p < 0.001). (Table 1). The early transmitral flow velocity (E) characterizing the early diastolic filling showed a significantly pronounced decrease in the case of hemodiafiltration (p < 0.001). In the case of hemodialysis E (p = 0.02) and E/A (p = 0.021), and in the case of hemodiafiltration E (p = 0.036) values positively correlated with changes in body weight, however regarding E/Ea and body weight a significant correlation was observed only in the case of hemodiafiltration (p =0.041) (Figure 2). Similar trends were found between E/Ea and the left atrial diameter. During hemodiafiltration the decreasing atrial diameter significantly correlated with the decrease in E/Ea (p = 0.013), while such a correlation was not observed during hemodialysis (p = 0.15) (Figure 3). Moreover, the starting diameter of the inferior vena cava showed a significant correlation with the E/Ea decrease but only in the case of hemodiafiltration (p = 0.009). The pre- and post-treatment left ventricular ejection fraction, left ventricular mass index, left ventricular end-systolic and end-diastolic diameters did not change significantly, and none of these parameters showed a statistically significant correlation (Table 1). During hemodiafiltration the RR cycle length on the ECG reading increased from the baseline after 30 minutes, which represents a decrease in the ventricular rate (p = 0.027). During the conventional hemodialysis a gradual decrease in the RR cycle length was observed, which, by the end of treatment had reached a significant level compared to the baseline (p = 0.024). Atrial fibrillation and malignant ventricular arrhythmias (ventricular tachycardia and ventricular fibrillation) were not observed in any of the treatments.

**Table 1:** The echocardiographic parameters measured during the study

	**HD**		**HDF**	
	**Before treatment**	**After treatment**	**Before treatment**	**After treatment**
**E (cm/s)**	103.4 ± 26.5	77.9 ± 22.6*	106 ± 26.5	72 ± 22*
**A (cm/s)**	92.2 ± 29.7	90.2 ± 26.2	95 ± 31	91.6 ± 22.8
**Ea (cm/s)**	8.6 ± 2.15	8.5 ± 1.95	12.4 ± 13.2	9.1 ± 2.25
**E/A**	1.37 ± 1.27	0.97 ± 0.63	1.2 ± 0.46	0.83 ± 0.34*
**E/Ea**	12.6 ± 3.55	9.88 ± 4.6*	11,65 ± 4.5	8,43 ± 4.46*
**LA (mm)**	45.4 ± 6.5	43.4 ± 6	45.1 ± 5,25	40.8 ± 5.8*
**EF (%)**	56.6 ± 9.2	56 ± 7.7	56,5 ± 8.7	54.6 ± 6.8
**LVMI**	203 ± 68.6	185 ± 63.7	180 ± 62.2	177.4 ± 64.7
**LVESD (mm)**	32.1 ± 7.2	31.6 ± 7.3	30.7 ± 5.55	30.6 ± 5.4
**LVEDD (mm)**	48.9 ± 7.8	47.5 ± 7.8	46.7 ± 6.6	45.8 ± 6.9
**VCI (mm)**	18.7 ± 2.8	16.2 ± 2.6*	19.7 ± 3	15.1 ± 2.8*

E and E/Ea decreased in both renal replacement modalities (p < 0.001), but E/A ratio’s significant decrease was observed only during hemodiafiltration.

HD: hemodialysis, HDF: hemodiafiltration, LA: left atrial cross diameter, EF: left ventricular ejection fraction, LVMI: left ventricular mass index, LVESD: left ventricular end systolic diameter, LVEDD: left ventricular end diastolic diameter, VCI: inferior vena cava diameter (*p < 0.05).

**Figure 4:**
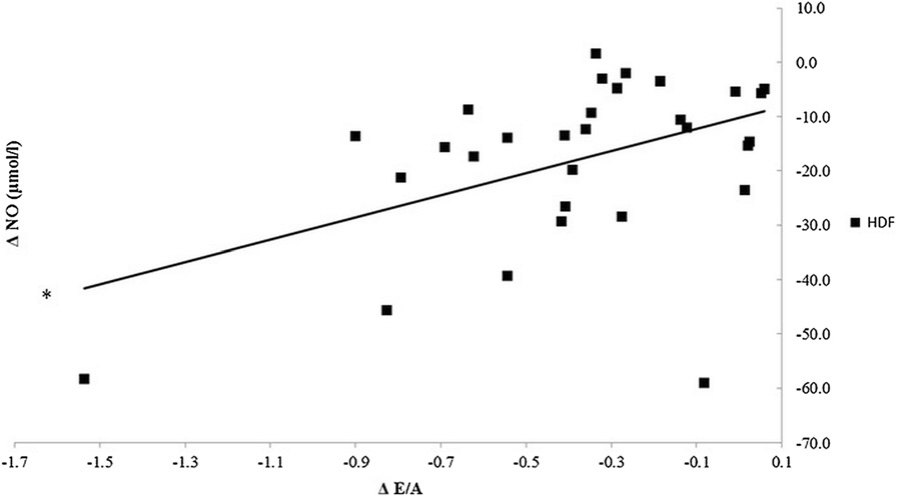
**Correlation between the change in nitric oxide concentration (ΔNO) and the decrease in the ratio between transmitral peak flow velocity and late diastolic transmitral velocity (ΔE/A) during hemodiafiltration.** NO concentration showed a positive correlation with the ratio between the early and the late diastolic flow velocities (E/A) (*p = 0.011).

Regarding blood pressure during both modalities a rapid decrease occurred after the initiation of the sessions, which, compared to baseline, reached a significant decrease after 15 minutes (p < 0.001). While the systolic and the diastolic values were higher even at the initial stages of the treatment in the hemodiafiltration group, this trend had no statistical significance. The systolic blood pressure did not change significantly afterwards, however, there was an increase in diastolic values. During both renal replacement therapies, there were no differences between the two modalities with respect to body weight and body mass index (BMI). The change in body weight was determined separately by gender, and it can be concluded that in both cases a significant reduction occurred (p < 0.001). During hemodiafiltration and conventional hemodialysis both NO and ADMA concentrations were observed to decrease significantly two hours after completion of the treatments (p < 0.001) (Table 2). However, the hemodiafiltration modality was associated with modulation in NO concentration that showed a positive correlation with the late diastolic transmitral flow velocity (A) (p = 0.02) and the ratio between the early and the late diastolic flow velocities (E/A) (p = 0.011) (Figure 4). Similar significant correlations were not observed during hemodialysis. Furthermore, ADMA level changes did not correlate significantly with the studied echocardiographic parameters.

**Table 2:** Nitric oxide (NO) and asymmetric dimethylarginine (ADMA) concentrations measured during the study

	**HD**		**HDF**	
	**Before treatment**	**After treatment**	**Before treatment**	**After treatment**
**NO(μmol/L)**	30.3 ± .76	12.23 ± 5.7*	29.4 ± 18.25	11.55 ± 5.54*
**ADMA(μmol/L)**	0.69 ± 0.2	0.55 ± 0.16*	0.64 ± 0.18	0.59 ± 0.15*

During hemodiafiltration and conventional hemodialysis both NO and ADMA concentrations were observed to decrease significantly two hours after completion of the treatments (*p < 0.001).

HD: hemodialysis, HDF: haemodiafiltration.

## Discussion

The mortality rate of patients participating in hemodiafiltration programs is 35% lower than those receiving conventional hemodialysis that may be attributed to multiple factors [9]. For instance, it has been demonstrated that in the case of hemodiafiltration the clearance of small- and medium- molecular weight substances (urea, creatinine, phosphate, beta-2 microglobulin, complement, leptin, cytokines, and homocysteine) was significantly higher compared to conventional hemodialysis [10-13]. Additionally, utilization of high-flux membranes enhances biocompatibility and more importantly, the concentration of acute phase proteins and inflammatory mediators (C-reactive protein, interleukin-1, interleukin-6, rheumatoid factor) do not rise during the treatment, or thereafter. By reducing the beta-2 microglobulin concentrations observed during hemodiafiltration, the incidence of amyloidosis can be reduced by approximately 50% [14-18]. Furthermore, by utilizing a high-flux dialysis membrane and an ultrasterile solution oxidative stress can be significantly mitigated which is directly associated with improvements in lipid profile [19,20]. On the other hand, NO (a crucial regulator of vascular tone) concentrations were shown to decrease during conventional hemodialysis due to its increased degradation and diminished NO synthase activity, altered ADMA concentrations and removal of NO metabolites [21]. Anemia is a common finding in hemodialysis patients with a prevalence of more than 80% and is frequently managed by erythropoietin (EPO) administration. Hemodiafiltration has been shown to improve anemia and it has been postulated that the suppression of inflammatory reactions and elimination of erythropoiesis inhibiting factors may contribute to the positive changes in the frequency of anemia [22]. Previous studies have shown that long-term uremic and hypovolemic status adversely affect left ventricular function [23]. The basis for that are the increased pre-, afterload, emergence of hypertension, left ventricular hypertrophy, coronary reserve lowering effect, and development of left ventricular systolic and diastolic dysfunction. Although the ventricular hypertrophy can be considered as an adaptive physiological response, it can result in myocyte-capillary mismatch and subsequent myocardial ischemia, fibrosis, as well as myocardial calcification [24]. Besides the numerous known factors, it might be also concluded that the patients receiving hemodiafiltration for their renal diseases were able to enjoy benefits resulting from the slower progression of the cardiac target organ damage as shown by Ohtake et al. [25]. Based on our results it can be stated that hemodiafiltration – even though there was no difference between the two modalities in terms of volume removal – decreased left atrial volume more effectively. While hemodiafiltration did not bring better improvement of E/Ea compared to hemodialysis, only in the case of hemodiafiltration could we find a positive correlation between E/Ea (representing the left ventricular pressure load) and body weight. The changes in the left atrial diameter during hemodiafiltration were closely related to the E/Ea decrease, whereas no such correlation was observed during conventional hemodialysis. This finding can be partly explained by the difference in tissue liquid distribution resulting from the benefits of the convective transport, which is the basis of hemodiafiltration. A degree of similarity between the volume removals might be indicated by the kinetics in the changes of blood pressure, but the systolic and diastolic data showed a non-significant difference even at the start of the treatment, which may affect the value of a comparative study. The decrease in NO and ADMA concentrations in both modalities might indicate similar mechanisms in the metabolism of vascular tone mediators. In contrast, significant correlations between NO and transmitral flow velocities (A and E/A) were observed only in the case of hemodiafiltration, which might attract attention to its beneficial effect on intracardiac pressure and left ventricular diastolic function. The analysis concerning the changes observed in the heart rate suggests a difference in the dynamics of volume removal in the case of the two modalities. Although atrial and ventricular arrhythmias were not observed in our study, the atrial and ventricular pressure load reduction during hemodiafiltration, through reducing the propensity for cardiac arrhythmias and arrhythmia vulnerability, can result in a favorable arrhythmia risk development. Our results show that the clinical and echocardiographic parameters demonstrated different data on several points, and that two renal replacement methods influenced the preservation of the left ventricular diastolic function differently. This might be the result of the more balanced, but increased intracardiac and intravascular volume reduction, the more effective balancing of the osmotically active agents’ concentration in the serum, which results in a more effective decrease in the left ventricular load pressure and the atrial diameter. Another plausible explanation is the potent antioxidant and anti-inflammatory effect, and also the more efficient removal of the small-and medium-molecular-weight materials and uremic toxins that could be observed during hemodiafiltration.

## Conclusions

This study investigated the differences between conventional hemodialysis and hemodiafiltration in the context of cardiovascular parameters. The strength of these findings stems from the fact that the same patients were examined by two different therapeutic modalities. Additionally, the obtained results did not show any correlation with the etiology of chronic kidney disease. Our results suggest that there were significant disparities between the studied echocardiographic markers and the clinical parameters representing the left ventricular diastolic function in the case of the two renal replacement modalities, despite the fact that there was no significant difference between the volume removals. These findings could be explained by the more efficient detoxification which occurs because of the convective transport present during hemodiafiltration, and also the differences between the dynamics of volume removal and its distribution among the liquid areas. Further studies are needed to analyze the impact of hemodiafiltration on ventricular functions in larger clinical trials.

### Study limitations

The number of the studied patients is relatively small, however all patients suitable for clinical investigations were enrolled from our center. A larger study population would help to clarify the role of different renal replacement therapies on left ventricular diastolic function. The determination of long term effects of hemodiafiltration on cardiac function was not possible during the present study, so further investigations are needed to get a clearer picture on this issue. Our results apply only to our selected group of patients without rhythm abnormalities and a low frequency of coronary artery disease.

## Competing interests

None of the authors have relationship with companies that may have a financial or non-financial interest in the information contained in the manuscript.

## Authors’ contributions

ÁC: He has participated in the design of the study, performed the statistical analysis, carried out echocardiographic examinations and drafted the manuscript. AP: She has participated in the design of the study and performed the statistical analysis, furthermore carried out echocardiographic examinations and helped to draft the manuscript. JK: He has participated in the design of the study and performed the statistical analysis, helped in the coordination and to draft the manuscript. KB: He has participated in the design of the study and performed the statistical analysis, and helped to draft the manuscript. AK: He has participated in the design of the study and performed the statistical analysis, and carried out echocardiographic examinations. GyP: He has participated in the design of the study, performed the statistical analysis, and helped to draft the manuscript. IL: He has participated in the design of the study and performed the statistical analysis, and helped to draft the manuscript. ZJ: He has participated in the design of the study and carried out echocardiographic examinations. AA: He has participated in the design of the study and helped to draft the manuscript. AZ: He has participated in the design of the study and helped to draft the manuscript. JB: He has participated in the design of the study and performed the statistical analysis, furthermore participated in the coordination and helped to draft the manuscript. ZSz: He has planned the design of the study and performed the statistical analysis, furthermore carried out most of the echocardiographic examinations. He took part in the coordination and drafted the manuscript. All authors read and approved the final manuscript.

## Pre-publication history

The pre-publication history for this paper can be accessed here:

http://www.biomedcentral.com/1471-2369/14/76/prepub
